# “Figuring out your place at a school like this:” Intersectionality and sense of belonging among STEM and non-STEM college students

**DOI:** 10.1371/journal.pone.0296389

**Published:** 2024-01-10

**Authors:** Sarah M. Ovink, W. Carson Byrd, Megan Nanney, Abigail Wilson

**Affiliations:** 1 Department of Sociology, Virginia Tech, Blacksburg, Virginia, United States of America; 2 Center for the Study of Higher & Postsecondary Education, University of Michigan, Ann Arbor, Michigan, United States of America; 3 TikTok/ByteDance, New York, New York, United States of America; 4 Global Squad, Hagerstown, Maryland, United States of America; The University of Alabama, UNITED STATES

## Abstract

**Background:**

Students’ *sense of belonging* in college—an individual’s feelings of contentment, mattering, importance, and “finding one’s place” in a social setting—can influence choice of major and career trajectory. We contribute to the belongingness literature through a mixed methods intersectional study of students attending a STEM-focused public university we call Meadow State University (MSU). We assess the potential for students’ intersecting social identities to differentially influence their experiences with *intersectional oppression*—subjection to multiple systems of oppression due to simultaneous membership in more than one marginalized group—that, in turn, may influence their college pathways. In addition, we explore whether intersectional differences affect sense of belonging differently in *STEM and non-STEM majors*. We employ a mixed-methods approach, informed by critical quantitative methods and in-depth interviews. We utilize quantitative institutional data measuring college satisfaction, expressed as “willingness to return” to the same university, for over 3,000 students during two academic years (2013–14 and 2016–17). Survey data explores college satisfaction as an indicator of intersectional differences in student experiences. Then, we analyze 37 in-depth interviews, collected between 2014–2016 at the same institution, to further contextualize the intersectional variation suggested by survey results.

**Results:**

Willingness to return is influenced by major, as well as academic, social, and campus belonging. Moreover, the extent to which these factors affected outcomes additionally varied by race/ethnicity, gender, family income, other background factors, and the ways these factors may intersect. Important components of academic belonging included faculty-student interactions, perceptions of academic support, and a privileging of STEM degree programs and students over non-STEM students and their degree programs at MSU. Faculty responsiveness and high impact practices like internships played an important role, particularly in STEM programs. Taken together, our findings demonstrate that, particularly for students of color and those subject to intersectional oppression due to multiple marginalized identities, satisfaction with academics did not always outweigh deficiencies in other areas of campus life shaping belongingness.

**Conclusions:**

Our mixed-methods approach contributes insights into how and why students’ background, individual choices, and institutional practices concurrently—and intersectionally—influence their ability to form a sense of belonging on campus. Structural changes are required to end practices that support intersecting systems of oppression by favoring White, upper-income men as the “default” STEM students in the U.S. Our research supports growing evidence that institutions must actively build models of inclusion for underrepresented and marginalized groups that address inequitable and unjust practices, providing transformative mentoring and educational guidance that attends to intersectional oppression, in order to effectively support the next generation of women and scholars of color.

## Introduction

Despite decades of attention to diversity in higher education, research consistently shows that cisgender women, low-income students, and those who identify as Black, Indigenous, and/or People of Color (BIPOC) continue to feel marginalized in U.S. colleges more often than cisgender men, middle to upper-income, and White students. Marginalization in the academy describes a range of experiences that stem from being a member of a numerically minoritized group (e.g., women majoring in mathematics), as well as exclusionary behaviors that can negatively affect minoritized group members’ academic trajectories (e.g., women in mathematics being interrupted, shut out of leadership positions, and/or subject to gender-based harassment) [1, 2]. Moreover, marginalization remains particularly acute in Science, Technology, Engineering, and Mathematics (STEM) fields [3–5]. Higher education research connects marginalization in college to students’ evaluations of *satisfaction* with their institutions, as well as variation in developing a strong *sense of belonging*. These related concepts refer to an individual’s feelings of contentment, mattering, importance, and “finding one’s place” in a social setting [6]. Satisfaction and sense of belonging in university settings vary by individual background, major, and other social factors, and previous studies demonstrate their importance for college outcomes [3, 6–10]. Recent social media campaigns like #BlackInTheIvory and #ShutDownSTEM brought attention to racism in the academy and scientific communities, calling on academics and scientists to push for equity and inclusion [11, 12]. Such efforts provide evidence that the opportunity to develop “belongingness” in academia is not universal [12], and that gendered, racialized, and socioeconomic inequalities within STEM fields remain an acute problem [13].

Past research documents multiple links between sense of belonging and positive college outcomes. For example, sense of belonging predicts students’ adjustment to college and intentions to persist [3, 6, 14], reduces probability of withdrawal [15]; and is associated with academic self-efficacy [16], academic and classroom engagement [17], as well as higher grades and satisfaction [6]. Nevertheless, there remain few theoretical or policy interventions effectively confronting the continued barriers to belongingness that marginalized students face. We argue this is because *intersecting* inequalities influencing development of sense of belonging go largely unexamined [18–23]. College experiences are shaped by systems of privilege and disadvantage that vary along intersections of gender, ethnoracial identity, and socioeconomic position including income and first-generation college-going status [24]. Yet, solutions to inequalities in the STEM pipeline often focus on a single axis of identity [23, 25]. For example, policies to diversify “high earning” STEM majors and improve BIPOC outcomes often overlook how BIPOC students’ barriers vary by gender and income [1, 20, 26]. Examining how college experiences differ intersectionally is vital for identifying institutional practices that can strengthen college belonging generally, and broaden STEM participation specifically.

We utilize mixed methods of inquiry and a case study approach to assess the potential for *intersectional oppression*—subjection to multiple systems of oppression due to simultaneous membership in more than one marginalized identity [2]—that differentially influences students’ college experiences and sense of belonging. We additionally examine whether and how different combinations of intersecting identities reflect sense of belonging differently for students pursuing *STEM and non-STEM majors*. Our setting is Meadow State University (MSU), a STEM-focused, predominantly White institution (PWI) in the Southeast region of the United States (all names are pseudonyms). In the section that follows, we review the extant literature connecting identity, experiences, and sense of belonging. We focus on literature that examines college contexts in the United States (U.S.), because our case is a U.S. university. We begin by reviewing the related concepts of *sense of belonging* and *satisfaction*, and include previous literature examining factors shown to influence their development. Our review focuses on two factors in particular: (1) intersectional oppression, and (2) how/why belonging develops differently in STEM and non-STEM majors. We then offer a guiding theoretical and conceptual framework for our study that is grounded in critical race theory (CRT) and critical quantitative methods.

### Sense of belonging and satisfaction

*Social connection* is a universal human need, facilitating access to resources and emotional support that sustain life [27]. *Sense of belonging*, as a form of social connection, refers to feelings of fitting in, feeling comfortable, establishing connections, being respected, safety, *mattering*, and *importance* in a social setting [8]. In short, sense of belonging acts as a “glue” connecting people to settings. As Strayhorn [6] writes, a sense of belonging “is a basic human need… [it] intersects with and affects social identities” (pp. 122–123). When belongingness is missing, individuals may not receive the same benefits from their experiences as those with a stronger sense of belonging [6]. Despite an emphasis on the individual level, previous research highlights how belongingness predictably varies due to power asymmetries shaping opportunities for engagement, resources, and social connections in a given setting.

It is important to note that the very concept of belongingness is burdened by the colonialist, White supremacist, and cisheteropatriarchal underpinnings of U.S. society in general, and the histories of many U.S. universities specifically. The historical residue from near-blanket exclusion of BIPOC mere decades ago still exists at universities that were literally built by enslaved people and shepherded indigenous genocide [28]. Thus, many universities remain “White spaces” [29]. Nevertheless, in recent years, even predominantly White institutions (PWIs) have engaged in myriad “diversity projects” intended to increase diversity, inclusion, and equity on campus; improving underrepresented and excluded students’ sense of belonging as a means of increasing representation, retention, and academic performance is a frequent goal [30].

Sense of belonging is a complex construct that can be examined at multiple levels: for example, belonging at higher education institutions [6, 16, 31], belonging in STEM generally [9, 32], and belonging in specific majors [33, 34]. One issue that must be confronted is the variety of ways that previous research has conceptualized sense of belonging. For example, Maton and co-authors discuss “sense of community” and “science identity,” [35] while Hazari and co-authors examine “performance/competence,” “recognition, “interest,” and “sense of belonging” as separate constructs [33]. Nunn [7], a sociologist of education, helpfully melds this constellation of constructs—which we argue are all connected to an overarching sense of belonging construct. In doing so, Nunn identifies three interrelated areas of belonging that matter for college students’ experience and outcomes: (1) Academic (feeling competent and comfortable in classes, labs, one’s major, etc.); (2) Social (connected to friends and social circles on campus); and (3) Community (feelings of inclusion across campus settings). Yet, limited research disaggregates sense of belonging to these three areas and examines them intersectionally [7, 18, 19, 21, 22].

Colleges and universities have long been interested in understanding the factors that can influence students’ judgment of how satisfied they are with their college experience [10]. Scholars have used multiple measures to assess satisfaction, aiming to capture students’ subjective assessments of the overall quality of their experience, including whether they have become “loyal” customers; that is, willing to return to the same institution [36–38]. In this study, we utilize a survey item designed to capture overall satisfaction (whether students would be “willing to return”) as an indicator of intersectional differences in student experiences. Students’ satisfaction, similar to their academic, social, and community sense of belonging developed throughout their college experience, remains unequal across multiple axes of difference. Areas of challenge include, but are not limited to: race/ethnicity [39], gender [40], and family income and associated factors, (e.g. paid work; first-generation status) [41]. For example, BIPOC students are less likely to express satisfaction and a sense of belonging, and students’ campus diversity climate perceptions are a contributing factor [3, 42]. Though women are graduating college at higher percentages than men, many academic majors remain gender-segregated, leading to corresponding satisfaction, belongingness, and career pathway differences [43]. For example, women, comprising 51% of the U.S. population, are overrepresented among agricultural and biology degree-holders (women earned 64% of these in 2020), yet remain underrepresented in physics (43%) and mathematical and computer sciences (26%) [44, 45]. Previous research has shown that identifying as a first-generation college student, as compared to those whose parents have completed college, is associated with: a lower-income background; less access to college-prep curriculum in high school; having received less guidance applying to and attending college from parents and high school personnel; and lower rates of college persistence, longer time to degree, and lower rates of completion [46–49]. We focus on three key axes of intersecting differences: race/ethnicity, gender, and family income and associated factors; yet, we acknowledge important sources of inequality that are out of our study’s scope (i.e., dis/ability status, sexuality, citizenship, and age).

### STEM inequalities

Reflective of institutions of higher education at large, the right to use and enjoy science—that is, what science looks like, who engages in science, and what science is used for—is not shared by all students, but rather, is unequally distributed due to intersecting inequities. The National Science Foundation considers the social sciences to be STEM fields; however, disciplines such as Psychology and Sociology report fewer inequalities as compared to so-called “hard” sciences (such as Physics and Math), and highly technical disciplines (such as Engineering and Computer Science). In the U.S., STEM has historically excluded women and people of color, reserving STEM identity and participation for White men, processes resulting in what Dancy et al. [50] term “science as White male property.” Thus, marginalized populations, including those who identify as BIPOC, low-income, ciswomen, and/or first in their families to attend college (first-generation), are less likely to attain STEM degrees despite comparable or higher aspirations to do so [51, 52], a pattern not found in other majors [22]. Among well-prepared BIPOC high school students, 44% of Black students desired STEM careers; however, just 27% of those interested actually attained STEM bachelor’s degrees [53]. This suggests that barriers within the STEM major-to-career pipeline disproportionately redirect marginalized students [25].

STEM career attainment is a social process, and the “desire of an aspirant is only one factor…An aspiring scientist relies on the judgment and invitation of practicing scientists throughout every phase of the educational and career process” [54] (p. 371). That is, inequities in *belongingness*, including faculty-student interactions, contribute to high attrition rates among populations marginalized in STEM [9, 23]. Further, STEM identity development and sense of belonging are intimately related [23, 55]. Previous research highlights how factors related to strong identity development such as mentorship, recognition, confidence, and interest are unequally experienced. For example, underrepresented students are less likely to have positive mentors like themselves in authoritative STEM positions, receive external recognition and validation of their performance and competence, and thus see themselves as scientists and feel like they belong [19, 23, 56]. These experiences reflect inadequate strategies to diversify the professoriate, effectively mentor BIPOC students, and connect them to STEM programs [18, 20]. Positive academic settings foster belongingness, including developing a “science identity,” while negative STEM climates essentially “weed out” marginalized students [57, 58].

Two survey-based studies complicate these conclusions. Nguyen and Riegle-Crumb [59] found that eighth-grade young women of color’s positive science-related counter-stereotypes (e.g., *disagreeing* with survey items communicating science stereotypes like “most scientists are geeks or nerds”) were associated with increased intent to major in computer science and engineering in the future. Verdin’s [23] survey of engineering majors at nine institutions affirmed that women of color were less likely to develop a sense of belonging, but found that *identifying* as an engineer was important to persistence while belongingness was not. Our study’s approach contributes to this complex set of narratives about what factors matter to the social process of STEM degree attainment by extending our inquiry to compare STEM and non-STEM students, and pairing students’ qualitative experiences with contemporaneous survey data.

STEM-focused ameliorative programs are increasingly found at U.S. universities. These programs, often designed for groups that experience marginalization in STEM, aim to improve students’ belongingness with and retention in STEM fields. Examples include summer bridge programs and other co-curricular instruction [60], faculty mentoring [20], living-learning communities [61], and near-peer mentoring [62]. However, research shows that such programs are often underutilized by women, BIPOC, and other marginalized populations, leading to differential academic outcomes [19, 56, 63]. This is in part because BIPOC and first-generation students are more likely to support family members or work for pay while attending college [22, 51, 53]. Hurtado et al. [53] document that while ameliorative efforts may help individuals, students participating in BIPOC-identified programming face social stigma because these programs do little to alter university cultures that assume White, male, affluent scientists are the norm. Thus, such programs may become a target for stereotypes from majority-group students, activating stereotype threat in participants [64].

### Theoretical and conceptual framework

In sum, the extant literature demonstrates that women, BIPOC, and low-income college students face barriers to equitable outcomes with men, White, and affluent students, and these problems are exacerbated in STEM fields [19, 22, 65]. However, many previous studies focus on one group at a time, or investigate facets of students’ identities and positions as additive variables [3]. A critical quantitative and intersectional framework allows us to examine how individual beliefs (meanings) and institutional practices (policies and programs) affect students’ sense of belonging across positions of difference, acknowledging the simultaneity of race-gender-class identities embedded within inequities [30, 66–72].

Critical quantitative and computational approaches aim to detail and expand engagement with experiences of marginalized and underrepresented communities. This area of methodological innovation is a burgeoning subfield in educational research and the social sciences that offers opportunities to refine our methodological approaches for improving STEM education [73]. Malik [74] defines critical quantitative and computational approaches as “the use of quantification and mathematical modeling (e.g., mechanistic, statistical, ‘algorithmic,’ simulation) within a critical and constructivist framework that understands quantification and modeling as social, situated, contingent, and ‘productive’ (often towards harm), not natural, universal, inevitable, or neutral” (p. 12). This orientation for quantitative research aligns with intersectionality’s emphasis that individuals cannot add or subtract facets of their lives [75]; therefore, an additive approach misses important nuances of human experiences. Hence, both sense of belonging and intersectional differences are difficult to assess quantitatively. Qualitative interviews can explore meaning and processes, but may lack generalizability. Mixed methods research promises to alleviate some of these concerns [76].

Intersectionality theory, rooted in critical race theory (CRT) and Black feminist theory, may be used as a guiding framework and set of analytical tools [2, 77–79]. In the realm of higher education, an intersectional framework pushes researchers to forefront how students of all backgrounds existing within the same institutional context nevertheless experience varying levels of privilege or marginalization; that is, students’ *social location* influences their collegiate experiences over and above academic ability and personal preferences [64]. In other words, one’s position at the intersection of race/ethnicity, gender, and income (among other statuses) remains important for determining whether an individual experiences advantage or oppression when navigating social systems, structures and institutions [79]. Moreover, an intersectional framework forefronts the complexity of social location, inviting researchers to consider how simultaneously experienced identities inflect experiences differently depending on context. For example, a student may experience belonging and advantage in pursuing a STEM major along one axis (e.g., identifying as a man), and experience disconnection and/or systemic oppression along other axes (e.g., identifying as Black and low-income).

We employ a mixed-methods approach, informed by critical quantitative methods and an intersectional framework, utilizing in-depth interviews to further contextualize data collected at an institution we refer to as “Meadow State University (MSU),” a large public research university. Survey analyses often cannot tell us *why* or *how* student experiences are linked to their sense of belonging. Thus, prior explorations of college belonging provide limited understanding of these complex, *intersecting* factors. We use MSU’s institutional survey data to explore the potential for intersectional differences in students’ reported college satisfaction and sense of belonging. Then, we analyze in-depth interviews conducted separately, at the same university, and during similar time periods as the survey data, to further contextualize the intersectional variation suggested by survey results. Our mixed-methods approach contributes insights into how and why students’ background, individual choices, and institutional practices concurrently—and intersectionally—influence their ability to form a sense of belonging on campus.

Utilizing survey data assessing student *satisfaction* and *sense of belonging*, and in-depth interviews exploring aspects of students’ *sense of belonging* in greater detail, we build on this literature to investigate: (1) How satisfaction varies by student identity, including race/ethnicity, gender, and their intersections; (2) How students’ satisfaction is influenced by academic, social and campus belonging, and other student characteristics such as working for pay, first-generation, and transfer status; (3) How college major (STEM/non-STEM) influences associations between race/ethnicity, gender, their intersections, and satisfaction; and (4) What qualitative interviews reveal about *why* satisfaction, factors influential for satisfaction, and sense of belonging vary by race/ethnicity, gender, family income and associated factors, major, and their intersections. Answering these questions advances our understanding of how, when, why, and for whom sense of belonging develops at MSU, a PWI that has publicly affirmed its commitment to diversity, inclusion and equity. Our findings point toward avenues for reducing campus inequalities with structural changes aimed at increasing equity in students’ belongingness.

## Materials and methods

Our mixed-methods approach begins with quantitatively exploring variations in three measures that correspond to students’ perceptions of their institution’s cultivation of academic, social, and campus belonging, and a reflective consequence of sense of belonging—students’ willingness to return to the same university (a proximate measure for student satisfaction)—using survey data collected at Meadow State, a large, public research university with a STEM emphasis. Then, we analyze in-depth interviews conducted between 2014–2016 with students attending the same institution to explore *how* and *why* satisfaction and sense of belonging vary, accounting for students’ complex experiences.

Our quantitative analyses use two academic years of data from the National Survey of Student Engagement (NSSE), an institutional survey asking students to evaluate their undergraduate institution. When developing our interview schedule, we obtained a list of variables included in MSU’s fielding of the NSSE survey. In this way, we were able to ascertain the extent to which NSSE’s survey questions would align with our interview questions, and to make sure that we asked certain questions that we knew we wanted to delve into in greater depth than surveys allow (e.g., “willing to return,” faculty interaction, and student income and employment questions). However, due to the long process involved in obtaining permission to use MSU’s institutional data for this study, we had no knowledge of the NSSE survey results prior to conducting interviews.

The NSSE data we use represent MSU students enrolled during the 2013–2014 (n = 1,864), and 2016–2017 (n = 1,528) academic years. Pooled together, these data allow for an intersectional examination across academic majors given the small sample sizes of BIPOC students each survey year. Pooled data contained 3,392 undergraduate students, with 2,272 (44.34%) enrolled in STEM majors and 2,852 (55.66%) in non-STEM majors. Our institutional data limit us to utilizing the *college* students’ major is housed in to indicate STEM or non-STEM affiliation. For example, both Psychology and Biology majors are defined as STEM by NSF, and they are also defined as such in our data because at MSU, both are housed in the College of Science. Despite the limitations of this organizational structure of degree programs at MSU shaping our data, we were able to capture a majority of STEM degree programs aligned with the following NSF STEM areas: (1) agricultural sciences; (2) biological sciences; (3) earth, atmospheric, and ocean sciences; (4) mathematics and computer sciences; (5) physical sciences; (6) social sciences; (7) engineering; and (8) health sciences.

The racial/ethnic composition of the sample was: 339 (9.83%) Asian or Pacific Islander (Asian/API), 89 (2.58%) Black, 93 (2.70%) Latine, 2,560 (74.20%) White, 369 (10.70%) Multiracial/ethnic or other race. Unfortunately, we are unable to examine Native American student experiences specifically, due to institutional aggregation of these students within the “multiracial/ethnic or other race” category available to researchers. We note that choosing a pan-ethnic term for people of Caribbean, Central and South American descent with Spanish-speaking heritage is politically and linguistically fraught. “Latinx” is common in academia, but just 3% of those it is intended to describe self-identify this way [80], and it is criticized as an Anglicization unpronounceable in Spanish. We use “Latine,” a gender-inclusive term increasingly used in Spanish-speaking lesbian, gay, bisexual, transgender, and queer (LGBTQ) communities [81].

The gender breakdown was nearly evenly split with 1,752 (51.47%) women and 1,652 (48.53%) men. NSSE did not provide a question to distinguish transgender and cisgender prior to 2013, nor does it include nonbinary options. Data for 2014 and 2017 included too few valid cases of non-binary students to be specifically examined in our analysis. Our qualitative interview protocol allowed students to freely identify their gender, but only one individual initially identified as trans/nonbinary. This individual later changed their designation, leaving us with no respondents who identified outside traditional binary categories.

Student composition by race and gender was: 1,331 (39.24%) White women, 1,207 (35.58%) White men, 152 (4.48%) Asian/API women, 186 (5.48%) Asian/API men, 47 (1.39%) Black women, 39 (1.15%) Black men, 46 (1.36%) Latine women, 47 (1.39%) Latine men, 170 (5.01%) Multiracial/multiethnic or “other race” (Multiracial/other-race) women, and 167 (4.92%) Multiracial/other-race men. Lastly, to maximize all possible available data for model estimates and the small sample sizes of students when considering race/ethnicity, gender, and academic major, we used multiple imputation with chained equations (MICE) to impute 10 sets of missing values to adjust for missing data on our four dependent variables and the academic and social background control measures before estimating our models [82, 83]. Missing data ranged from less than one percent to slightly above two percent for variables included in our models.

### Measures

#### Institutional cultivation of sense of belonging

Although the NSSE did not include explicit measures of students’ sense of belonging until 2020, previous administrations of the survey included a series of measures that arguably reflect students’ perceptions of how well their university cultivated academic, social, and community (campus) belonging, following Nunn’s [7] conception. Students were asked how much MSU emphasizes “providing support to help students succeed academically;” and “using learning support services (tutoring services, writing center, etc.)” (1 = very little; 2 = some; 3 = quite a bit; 4 = very much). These two measures formed a proximate measure for how well MSU cultivates *academic belonging* among students (α = .7663). Students were also asked how much MSU emphasizes “providing support for your overall well-being (recreation, health care, counseling, etc.);” “helping you manage your non-academic responsibilities (work, family, etc.);” and “providing opportunities to be involved socially.” These measures formed our proximate measure for how well MSU cultivates *social belonging* among students (α = .7944). Lastly, students were asked how much MSU emphasizes “attending events that address important social, economic, and political issues;” “encouraging contact among students from different backgrounds (social, racial/ethnic, religious, etc.);” and “attending campus activities and events (performing arts, athletic events, etc.)” These three measures formed our proximate measure for how well MSU cultivates *campus belonging* among students (α = .7678). Table 1 provides a summary of the scale construction of the three forms of sense of belonging and model fit of the confirmatory factor analysis, which provided a moderately strong fit with the proximate measures available in the NSSE.

**Table 1:** Construction of academic, social, and campus belonging scales.

Scale Item	Factor Loading	α
*Academic belonging*		.766
MSU emphasizes “providing support to help students succeed academically.”	.819	
MSU emphasizes “using learning support services (tutoring services, writing center, etc.).”	.771	
*Social belonging*		.794
MSU emphasizes “providing support for your overall well-being (recreation, health care, counseling, etc.).”	.740	
MSU emphasizes “helping you manage your non-academic responsibilities (work, family, etc.).”	.680	
MSU emphasizes “providing opportunities to be involved socially.”	.777	
*Campus belonging*		.767
MSU emphasizes “attending events that address important social, economic, and political issues.”	.716	
MSU emphasizes “encouraging contact among students from different backgrounds (social, racial/ethnic, religious, etc.).”	.700	
MSU emphasizes “attending campus activities and events (performing arts, athletic events, etc.).”	.687	
Χ^2^		702.472***
RMSEA		.104
SRMR		.040
CFI		.941

* *p* < .05

** *p* < .01

*** *p* < .001.

#### Willingness to return

Students were asked whether, if they could start their undergraduate studies over, would they attend the same institution again (1 = definitely no; 4 = definitely yes). Referring to it as “willingness to return,” this measure captures institutional *satisfaction* as defined previously, which we use as an indicator of intersectional differences in student experiences [36–38]. The extent to which students of differing backgrounds, identities, and majors express a willingness to return signals whether the institution is perceived as supportive and inclusive. Disparities in cultivating satisfaction, in relation to belonging, reveal for whom the institution falls short. However, students could also interpret the question as a cost/benefit analysis. Thus, relying solely on survey items could lead us to miss important nuances of belongingness. Our mixed methods approach mobilizes in-depth interviews to address this limitation, allowing us to explore whether students’ willingness to return is predicated on feelings of belongingness not captured by available survey questions.

#### Academic and other social background characteristics

We included a group of academic and other social background characteristics of students as control measures for exploring inter and intracategorical intersectionality among students. Among the academic characteristics, we included academic year (1 = freshman; 2 = sophomore; 3 = junior; 4 = senior). Unclassified students were omitted (n = 54). Self-reported average grades were also included. While the NSSE uses an 8-point scale ranging from “1” equaling “C- or lower” and “8” equaling “A”, we decided to rescale the measure to better align with a 4.0 grading scale where each unit increased by .3 (1.7 = C- or lower; 4.0 = A). We also included two measures of students’ work experience on- and off-campus (each measured: 1 = 0 hours; 8 = 30+ hours). In some analyses, we examine STEM (1) and non-STEM majors (0) separately.

In relation to the other social background characteristics of students beyond their ethnoracial and gender categories (each operationalized as dichotomous identifying variables), we identified if students transferred to the university (1 = yes; 0 = no). Available measures associated with family income included self-reported first-generation status, defined as when neither parent or guardian has a BA/BS degree (1 = yes; 0 = no), and students’ average weekly hours worked for pay on and/or off campus (1 = 0 hours; 8 = 30+ hours). Lastly, we control for the year the NSSE was administered (1 = 2013–14; 2 = 2016–17). Table 2 reports descriptive statistics for the MSU NSSE sample.

**Table 2:** Descriptive statistics: NSSE students at Meadow State.

Variable	Mean	SD	% Missing
*Institutional cultivation of sense of belonging*			
Academic belonging	3.01	.76	1.89
Social belonging	2.92	.71	2.06
Campus belonging	2.76	.73	2.03
*Willingness to return*			
Select same institution to pursue degree	3.52	.72	.85
*Academic major*			
STEM major	.65	.48	.00
*Race/ethnicity*			
Asian/API	.10	.30	.00
Black	.03	.16	.00
Latine	.03	.16	.00
White	.74	.43	.00
Multiracial/ethnic and other race	.10	.30	.00
*Gender*			
Women (1 = yes)	.51	.50	.00
*Other academic and social background characteristics*			
Academic year (1 = freshman; 4 = senior)	2.72	1.38	1.83
Grades (1.7 = C- or lower; 4.0 = A)	3.36	.54	.27
Transfer student (1 = yes)	.16	.37	.15
First-generation student (1 = yes)	.20	.40	.44
Total hours worked on-campus for pay during year (1 = 0 hours/week; 8 = 30 or more hours/week)	2.70	.02	1.53
Total hours worked for pay off-campus during year (1 = 0 hours/week; 8 = 30 or more hours/week)	1.81	.02	1.47

### Quantitative methods

Our analyses of NSSE data seek to answer our first three research questions, documenting variation in students’ perceptions of how well MSU cultivates the three forms of sense of belonging identified by Nunn [7] and their willingness to return to MSU. We conducted linear regressions with fixed effects to examine changes in students’ perceptions of their institution’s cultivation of sense of belonging and their willingness to return to MSU. Similar to López et al. [69], we utilize a dual strategy to explore students’ intersectional social locations, which are associated categories of race/ethnicity, gender, and academic major that represent “categories of experience in a given sociohistorical and political economic context” such as on college campuses and society more broadly [55]. First, we estimated models for each of our four dependent variables that included students’ race/ethnicity, gender, and academic major separately and with interaction effects between these three key features of students’ social locations. White students, women, and non-STEM majors were reference groups in these models. Next, we estimated fixed effects models that were fully saturated, meaning the models included all combinations of ethnoracial and gender groupings embedded within students’ academic programming (20 social locations) in our analyses, with white men in STEM programs as the reference group given their historical privileging at the university. Through this analytic approach, we can explore inter- and intracategorical intersectionality or the between-group differences in students’ perception of MSU’s cultivation of academic, social, and campus belonging among students and their willingness to return to MSU, and further examine the qualitative differences between students in STEM and non-STEM majors [69, 77].

### Qualitative methods

In 2014, we began recruiting MSU undergraduate students for a longitudinal study of intersectional differences in college-to-career trajectories. We utilized email, flyers, in-person presentations across majors, and word of mouth. To increase BIPOC participation, we contacted organizations such as the Black Student Union and “Latines in STEM.” Our recruitment and longitudinal interview protocols necessitated collecting and utilizing identifying information about individual participants. We obtained research protocol and ethics approval from Virginia Tech’s Human Research Protection Program for all of our research activities that involved human subjects. We note that students may have been included in both NSSE survey waves if they chose to respond, and may have also been included in our qualitative sample. However, we are unable to identify if the same student was included in multiple samples because of the deidentification of the quantitative data required by the institution in providing access to the authors.

By July 2014, we had amassed a pool of over 400 potential interviewees, using an initial short survey that collected basic demographic information, as well as willingness to complete multiple waves of interviews over five years. Along with a team of six undergraduate and graduate research assistants, the first author developed an interview schedule (see supplemental materials). We piloted the interview schedule with each other, as well as with nine undergraduate volunteers with a variety of majors and racial/ethnic identities, who were previously unconnected to our research team.

After obtaining external funding (see funding statement), our team transitioned to selecting interviewees from among the general pool of volunteers. We were selective, in order to create a corpus of interviewees that were diverse in terms of gender, racial/ethnic identity, and major. The first author was responsible for training graduate and undergraduate students to conduct, code and analyze interviews, based on her years of experience with interviews as a primary research method. Interviewers used the same interview schedule with every respondent, though we utilized a semi-structured format that allowed for variation in follow-up questions based on interviewee responses. For example, not all interviewees reported facing “challenges” because of their identities, but every respondent who did face a challenge was invited to tell us about those experiences in detail. All first-wave interviews used in this study were coded twice, with the second coder providing a “check” on the first. All disagreements in coding resulted in a meeting to reconcile coding; coders discussed any differences, sometimes with the first author as well, until agreement was reached.

Our efforts resulted in 113 first-wave interviews, completed between 2014–2016. Respondents included 54 men (47.8%) and 59 women (52.2%). Forty (35.4%) identified as BIPOC, and 55 (55.6%) were STEM majors. Our sample also included two STEM/STEM double majors, and five STEM/Non-STEM double majors. Our team, including graduate and undergraduate researchers, spent several months considering the size and composition of the sample to include for the qualitative portion of the present article. It is important to note that our goal in including qualitative interviews is not to be able to generalize to the overall population of MSU students, but to contribute in-depth analysis that is purposeful, theoretically driven, and that complements our quantitative analyses. Following Patton [84], our team utilized the logic of theory-driven purposeful sampling to select 37 “information-rich cases for study in depth” (p. 168).

In selecting cases, we sought representation among four ethnoracial identities: White, Black, Asian/API, and Latine. In choosing which cases to include in order to offer in-depth comparisons, we (a) selected among those respondents who provided information-rich interviews (some interviewees were not as forthcoming); (b) oversampled groups that remain underrepresented at MSU so that we could explore experiences of those existing at the intersections of marginalized racial/ethnic-gender identities while (c) slightly oversampling STEM majors among marginalized groups, to be able to investigate whether and how different combinations of intersecting identities reflect sense of belonging differently for students pursuing STEM and non-STEM majors, a key goal of the present article. We note areas for future research with this corpus of interview data—including exploring subsequent waves of interviews—in our conclusion.

Among these 37 information-rich cases, twenty identified as men (54.1%), 17 as women (45.9%). Eight (21.6%) identified as White, 12 (32.4%) as Black, 4 (10.8%) as Latine, 12 (32.4%) as Asian/API, and one as multiracial. Interviewees also estimated their family’s income range, and reported the highest degree attained by each parent or guardian. Based on direct reports of parents’ and guardians’ earnings, as well as observed patterns in qualitative responses—including whether respondents self-identified as struggling or “sacrificing” to afford MSU and/or living in its comparatively high-cost college town—we report these as “low-income” (<$50,000; 19%) and “middle/high-income” (>$50,000; 81%). Over half were STEM majors (22; 59.5%), reflecting its predominance at MSU. Table 3 compares the study samples and the MSU population as it was in the 2014–2015 academic year.

**Table 3:** Demographic comparisons^1^.

Demographics^1^	NSSE Sample (n = 3,392)	Meadow State^2^ (n = 24,191)	Wave 1 Analytical Sample (n = 37)	Wave 1 (n = 113)
*Race*				
White	74.2%	69.7%	21.6% (8)	38.9% (44)
Black	2.6%	3.6%	32.4% (12)	16.8% (19)
Latine	2.7%	5.3%	10.8% (4)	9.7% (11)
Asian/API	9.8%	9.3%	32.4% (12)	25.7% (29)
Multiracial/Other	10.7%	12.1%	2.7% (1)	8.8% (10)
*Gender*				
Men	48.5%	41.9%	54.1% (20)	47.8% (54)
Women	51.5%	58.1%	45.9% (17)	52.2% (59)
*Major* ^3^				
STEM	44.3%	57.3%	59.5% (21)	55.6% (55)
Non-STEM	55.7%	42.7%	40.5% (16)	44.4% (44)
*Academic year*				
First-year	36.8%	24.5%	19% (7)	25.7% (29)
Second-year	2.8%	19.7%	19% (7)	15% (17)
Third-year	11.7%	24.1%	32.4% (12)	25.7% (29)
Fourth-year	48.6%	31.7%	21.6% (8)	28.3% (32)
Fifth-year	-	-	8% (3)	5.3% (6)
*Income status*				
Low-income	19.6%	16.5%	19% (7)	18.6% (21)
First-generation		17.6%	19% (7)	25.7% (29)

1. Wave 1 2014–2016; NSSE pooled data AY 2013–14 and 2016–17; MSU data AY 2014–2015.

2. Data from MSU’s Office of Institutional Research.

3. Excludes double majors.

Interviews were semi-structured, and ranged in length from 30 minutes to over two hours. Interview length varied based on respondents’ experiences (some had more to relate than others) as well as individual verbosity. Transcripts of our 37 illustrative cases were coded using Dedoose software. Utilizing an iterative process and multiple coders to enhance reliability, we completed two rounds of coding. After each interview had been coded twice by two different coders, the two coders met to discuss their findings collaboratively, and to “reconcile” any differences in their application of the codebook, a process inspired by the “negotiated agreement” approach [85]. Any remaining disagreements were resolved via group discussions with the first author. In this way, we negotiated 100% agreement on all coding decisions.

Open coding resulted in preliminary, *descriptive* coding exploring the scope of students’ experiences. For example, we coded for “educational decisions,” developing a typology of motivations (“economic returns;” “financial cost”) from interviewee responses. Second-round coding included a more focused examination in order to develop *thematic* codes related to our *sensitizing concepts*, a term referring to theory-based ideas that provide focus and direction for qualitative analyses [86]. Examples of sensitizing concepts for these analyses include “Networking” and “Underrepresented Identity Benefits/Challenges.” Our analysis proceeded with attention to themes salient to students’ satisfaction and sense of belonging; how interactions with faculty, academic characteristics, and other social factors relate to belongingness; and evidence of intersectional differences therein. For example, thematic codes such as “getting to know professors” helped us identify intersectional differences in how, when, and why students interact with or avoid professors.

In analyzing our 37 illustrative cases, we sought to answer our final research question for the present article: *why* satisfaction, and factors influential for satisfaction, vary by race/ethnicity, gender, income, major, and their intersections. Ultimately, students’ responses to our open-ended “willingness to return” interview question indicated *sense of belonging* as a prominent explanation.

## Results

### Quantitative analyses

Table 4 presents the fixed effects models for students’ perceptions of institutional cultivation of the three forms of sense of belonging based on Nunn’s [7] conceptualization. We additionally conceptualize “willingness to return” to MSU if students could “do it all over again” as an extended consequence of sense of belonging and satisfaction with the college experience. These models utilize the first component of our dual modeling strategy based on López et al. [69] of incorporating interaction effects between students’ race/ethnicity, gender, and academic major (STEM or Non-STEM) to identify baseline intersectional differences in perceptions of institutional cultivation of sense of belonging. To facilitate conversations about magnitude of impact, we report the average marginal effects.

**Table 4:** Intersectional fixed effects models of academic, social, and campus belonging and willingness to return to MSU with interaction terms.

Variable	Academic Belonging			Social Belonging			Campus Belonging			Willingness to Return		
	AME	SE	*p*	AME	SE	*p*	AME	SE	*p*	AME	SE	*p*
Men	-.188***	.052	.000	-.156**	.048	.001	-.115*	.048	.018	-.218*	.049	.012
Asian/API	.032	.084	.703	.017	.079	.824	.096	.079	.226	-.156*	.071	.030
Black	.053	.153	.728	.032	.126	.795	-.019	.131	.880	-.199	.139	.153
Latine	-.100	.159	.528	.081	.127	.521	.113	.146	.438	-.016	.156	.915
Multiracial/other-race	.019	.080	.806	-.043	.069	.533	.125	.077	.104	.008	.083	.915
STEM major	.131*	.061	.032	.035	.056	.527	-.020	.060	.735	.067	.060	.133
*Race-gender interactions*												
Asian/API men	-.151	.168	.367	-.203	.148	.170	-.236	.149	.114	-.209	.141	.139
Black men	-.529	.314	.093	-.485*	.238	.041	-.350	.253	.166	.482	.258	.061
Latine men	-.097	.313	.756	-.183	.249	.462	-.031	.283	.912	-.380	.302	.208
Multiracial/other-race men	-.069	.157	.660	.074	.134	.578	.043	.149	.769	.080	.170	.638
*Gender-STEM interaction*												
Men and in STEM	.008	.083	.918	-.042	.077	.583	-.004	.082	.959	.151	.090	.094
*Race-STEM interactions*												
Asian/API students in STEM	-.221	.171	.195	-.149	.159	.351	-.170	.164	.300	.120	.136	.378
Black students in STEM	-.171	.300	.569	-.227	.256	.375	-.390	.272	.152	-.285	.287	.321
Latine students in STEM	.145	.313	.642	.122	.244	.617	-.027	.295	.925	-.125	.321	.696
Multiracial/other-race students in STEM	.239	.163	.143	-.034	.145	.811	-.172	.162	.289	.008	.168	.958
*Race-gender-STEM interactions*												
Asian/API men in STEM	-.150	.226	.506	.210	.207	.310	.027	.201	.891	.556**	.206	.007
Black men in STEM	-.389	.462	.399	-.518	.321	.107	-.346	.366	.344	.106	.373	.776
Latine men in STEM	.274	.440	.534	.349	.355	.325	-.231	.433	.593	-.877	.493	.076
Multiracial/other-race men in STEM	.007	.240	.975	-.445*	.218	.041	-.461	.237	.052	-.040	.265	.879
Fixed Effects	Yes			Yes			Yes			Yes		
Interclass correlation (ρ)	.465			.467			.456			.454		
F	3.14***			4.85***			3.03***			2.91***		

Note: Analyses used imputed data and contained 3,392 cases each. Control measures not shown include academic year of students, reported grades, whether the student transferred to MSU, first-generation status, hours reported working for pay on- and off-campus, and year of survey. White students, women, and non-STEM majors are reference categories. “AME” represents average marginal effects. “SE” represents standard errors.

* *p* < .05

** *p* < .01

*** *p* < .001.

When considering students’ race, gender, and academic major separately, men were less willing to return to MSU compared to women (AME = -.218, *p*<. 05). Moreover, men held lower levels of academic belonging (AME = -.188, *p*<. 001), social belonging (AME = -.156, *p*<. 01)., and campus belonging (AME = -.115, *p*< .05) than women at MSU. Asian/API students also had less willingness to return to MSU compared to white students (AME = -.156, *p*<. 05). STEM majors reported more positive perceptions of MSU’s cultivation of academic belonging than non-STEM majors (*b* = .131, *p*< .05).

Turning to the interaction terms included in the models, we find a somewhat mixed pattern of results among racially minoritized students. Few interaction terms across race/ethnicity, gender, and academic major reached statistical significance in our initial models. When considering the intersection of race and gender, Black men reported lower perceptions of social belonging on campus (AME = -.485, *p*<. 05). Asian/API men who were STEM majors reported higher levels of willingness to return to MSU (AME = .556, *p*<. 01). However, multiracial/other race men in STEM reported lower perceptions of social belonging on campus (AME = -.445, *p* < .05). These initial findings provide a complicated picture of how social location—including students’ race/ethnicity, gender, and academic major—relates to three forms of belongingness and willingness to return to MSU.

The second component of our quantitative analyses tests fully saturated fixed effects models. Table 5 considers students’ responses with regard to 20 intersected social locations. Here, we seek to uncover students’ inter- and intracategorical intersectional experiences, as compared to White men in STEM majors, the group historically most privileged at MSU. As with the models presented in the previous table, our findings in Table 5 suggests a mixture of positive and negative trends in terms of MSU’s cultivation of sense of belonging, and students’ willingness to return to MSU.

**Table 5:** Intersectional fixed effects models of academic, social, and campus belonging and willingness to return to MSU by social location.

Variable	Academic Belonging			Social Belonging			Campus Belonging			Willingness to Return			N
	AME	SE	*p*	AME	SE	*p*	AME	SE	*p*	AME	SE	*p*	
STEM White men (reference)	----	----	----	----	----	----	----	----	----	----	----	----	886
Non-STEM White men	-.026	.094	.775	.031	.090	.729	-.061	.092	.505	-.154	.102	.134	321
STEM White women	.286***	.074	.000	.218**	.071	.002	.073	.069	.292	.112	.065	.085	748
Non-STEM White women	-.000	.082	.997	.086	.077	.267	.102	.082	.217	.060	.077	.437	583
STEM Asian/API men	-.023	.135	.862	.044	.119	.710	.004	.109	.970	-.078	.128	.541	143
Non-STEM Asian/API men	.117	.202	.562	-.180	.186	.333	-.039	.185	.834	-.646***	.180	.000	43
STEM Asian/API women	.072	.167	.665	-.006	.147	.967	.094	.148	.526	-.172	.144	.234	95
Non-STEM Asian/API women	.361	.213	.090	.481*	.209	.021	.451*	.229	.049	.117	.143	.413	57
STEM Black men	-.268	.302	.376	-.305	.237	.198	-.307	.245	.211	.067	.239	.778	22
Non-STEM Black men	.122	.357	.731	.215	.250	.389	.041	.301	.890	-.037	.276	.893	17
STEM Black women	.405	.335	.227	.372	.218	.089	.008	.192	.966	-.684**	.209	.001	29
Non-STEM Black women	.358	.229	.119	.307	.312	.325	.420	.370	.257	-.043	.350	.901	18
STEM Latine men	.013	.290	.964	.175	.248	.480	.065	.274	.810	-.434	.316	.169	34
Non-STEM Latine men	-.255	.351	.468	-.165	.278	.552	.306	.346	.376	.449	.394	.255	13
STEM Latine women	.012	.311	.967	.188	.238	.430	.217	.238	.363	.440*	.223	.049	23
Non-STEM Latine women	.009	.330	.977	.312	.239	.192	.088	.349	.801	-.115	.362	.749	23
STEM Multiracial/ethnic and other race men	.087	.126	.490	-.038	.114	.737	.049	.125	.695	.068	.147	.641	125
Non-STEM Multiracial/ethnic and other race men	.072	.205	.724	.396*	.200	.047	.499*	.214	.020	.100	.243	.680	42
STEM Multiracial/ethnic and other race women	.307	.177	.083	.155	.133	.243	.191	.143	.182	.014	.146	.922	106
Non-STEM Multiracial/ethnic and other race women	-.146	.145	.313	-.190	.150	.205	.097	.171	.571	-.035	.165	.828	64
Fixed Effects	Yes			Yes			Yes			Yes			
Interclass correlation (ρ)	.463			.465			.452			.455			
F	3.13***			4.41***			2.63***			2.51***			

Note: Analyses used imputed data and contained 3,392 cases each. Control measures not shown include academic year of students, reported grades, whether the student transferred to MSU, first-generation status, hours reported working for pay on- and off-campus, and year of survey. White men in STEM degree programs were the reference category. “AME” represents average marginal effects. “SE” represents standard errors.

* *p* < .05

** *p* < .01

*** *p* < .001.

In comparison to White men in STEM, White women in STEM majors (AME = .286, *p*< .001), held more positive views of MSU’s cultivation of academic belonging. Considering MSU’s cultivation of social belonging, again, White women in STEM (AME = .218, *p*< .01) held more positive views compared to White men in STEM majors. Asian/API women in non-STEM majors (AME = .481, *p*< .05) and Multiracial/other race men in non-STEM majors (AME = .396, *p*< .05) also held more positive views of social belongingness. Further, Asian/API women in non-STEM majors (AME = .451, *p*< .05) and Multiracial/other-race men in non-STEM majors (AME = .499, *p*< .05) held more positive views of MSU’s cultivation of campus belonging as compared to White men in STEM. Turning to students’ willingness to return, Latine women in STEM majors (AME = .440, *p*< .05) were more willing to return compared to White men in STEM majors. However, Asian/API men in non-STEM majors (AME = -.646, *p*< .001) and Black women in STEM majors (AME = -.684, *p*< .01) reported less willingness to return compared to White men in STEM majors.

We were secondarily interested in directly exploring associations between our set of control measures—academic year, grades, being first in one’s family to attend college, being a transfer student, working for pay, and survey year—and the three forms of belonging as well as willingness to return. We find that as students’ time on campus increases, as measured by their academic year, all three forms of belonging decrease. Higher grades are associated with higher academic, social, and campus belonging. As expected, being a transfer student was associated with decreased social and campus belonging. Interestingly, academic and social belonging, as well as willingness to return, appear to be decreasing over time, based on NSSE survey year. Students who worked more hours on campus for pay corresponded to increased social and campus belonging, and increased willingness to return, but did not relate to academic belonging. Similar findings did not exist for hours worked at off-campus jobs. How such work may relate to sense of belonging and degree pathway obstacles such as certain majors having hidden costs, and these other connections are presented in this article’s supplementary information (S1 Table), and helped inform our interpretations of the findings we explore in greater depth in our qualitative analyses.

Though small numbers of respondents in some of the race-gender-major groupings warrant cautious interpretations, it is important to recall these reported patterns represent meaningful differences in student experiences. Our dual modeling strategy adds nuance to our understanding of how much students felt MSU cultivated academic, social, and campus belonging. The mixed results from our analyses suggest potential gaps remaining between institutions’ attempts to cultivate belonging, and students’ reflections on whether, in the end, they felt fully satisfied with their college educational experience.

Rather than assume additive interpretations, our analyses highlight how students are differentially experiencing STEM and non-STEM programs at MSU in ways that demonstrate a complex picture of inter- and intracategorical intersectionality [69, 77]. Lastly, the similarity in the size of F and the interclass correlation (ρ) for the models suggest the dual modeling strategy of complimentary interaction terms and full saturation with students’ social locations uncovers experiences hidden beneath the traditional multiplicative approach to quantitative intersectionality analyses [69, 87].

### Qualitative analyses

We use 37 in-depth interviews drawn from the same institution as our survey data to triangulate these patterns, exploring *how* and *why* observed quantitative patterns might occur. We utilize interviewees’ stories to investigate the relationships between gender, ethnoracial identity, major, and satisfaction. Then, we examine how student identity and major further inflect experiential factors suggested by prior research and our survey data to affect willingness to return: faculty interactions, institutional support, and working for pay. Our dual aims are to add (1) *depth* to our quantitative examination of group differences and influential social factors; and, (2) *nuance* by exploring factors not available in NSSE, such as a direct measure of family income. We find that intersectional differences in *sense of belonging*—or lack thereof—was a prominent explanation for respondents’ willingness to return.

### Qualitative insights: Willingness to return

Eight interviewees (21.6%) would not return to MSU if they could choose again. Some patterns identified in survey data surfaced in interviews, such as a greater number of men (n = 5) unwilling to return than women (n = 3). Non-STEM majors were in the majority among interviewees unwilling to return (n = 5: two Black men; one Black woman; and two White men, one of whom was first-generation). All STEM majors unwilling to return identified as BIPOC (n = 3: one Black woman, also a transfer student; one Asian woman; and one Latine man).

Non-STEM majors unwilling to return perceived less prestige in, and support for, non-STEM subjects at MSU. Ryan, a low-income White man and first-generation scholar who switched from Engineering to English, felt stigmatized: “I have been embarrassed to tell people what my major is.” English suited Ryan’s career goals, but he lamented that “people often assume that like, ‘Oh you shifted to English because you were too stupid to do the other thing,’ or because, like, you are impractical or lazy.” Kyle, a Black English major, wished he had instead attended MSU’s rival institution, because of its superior liberal arts reputation. The stereotype of the “unemployable” English major is widespread in the U.S., so similar sentiments may be found at other universities. However, MSU’s reputation as a STEM-centric institution, where Engineering is held in particularly high regard, likely exacerbated these respondents’ negative feelings about their majors. Unwillingness to return under these circumstances may be particularly elevated among those who, like Ryan, switch from a high- to a low-prestige major.

A commonality among non-STEM BIPOC unwillingness to return was MSU’s lack of diversity, which inhibited social and campus belonging. Hayden, a Black Sociology major, reported that the “White-centric focus” at MSU caused problems. He resented that “the way people tend to go about it is to try to make it the responsibility of the Black student to fix the problems with race on this campus. I came here to get a degree; who came here to fix the problems on this campus? I don’t get paid for that!” Non-STEM BIPOC students experienced interlocking disadvantages when they felt coerced to help “fix” hostile environments.

Though no STEM majors reported pressure to “fix the problems with race” at MSU, and were happier with the level of academic support, STEM programs did not protect marginalized students from regularly experiencing microaggressions. Rachel, a Black woman in Engineering, participated in several programs supporting Black students. Yet, when asked what it takes to “fit in” at MSU, she paused for a long time before answering, “being White? I don’t know, having straight hair?” She recounted how some White peers enviously pointed out her “permanent tan,” while others denigrated her “gross” haircare routine. Rachel straightened her hair one semester to fit in more, because, as she put it, “I hate being the center of attention, and my hair made me feel like that. Along with my skin color.” She tired of her “uncomfortable” MSU life: “It does affect me personally…I’m trying to find myself and be true to who I am, but at the same time it really takes a toll on your self-confidence.” Rachel thought, “nobody would find me attractive.” Rachel disavowed gender as influencing her experiences, saying “I have no problems being a woman…I am Black first, then a woman;” yet, the microaggressions she reported mainly concerned beauty standards that are simultaneously gendered *and* racialized. Rachel appreciated MSU’s academic opportunities but regretted not choosing a college with a more diverse student population. As she put it:

Rachel: I feel better if either a) people were all Black, or b) people were all different. There is not enough differences here for me to feel comfortable. I would rather it just be all different. I feel better when it’s all different.

Interviewer: So if there is a more diverse population here you will feel much better?

Rachel: Yeah.

Rachel wanted a more diverse student body so that she could be herself, without having to explain or call attention to herself—something she felt was impossible at MSU.

Sharlyne, a Black woman, was satisfied with her Nutrition major, but not the “forced community” of MSU’s social environment. Asked what it takes to “fit in,” she responded: “I would say if somebody wanted to fit in at Meadow State, they’d be White. They’d have money. They’d probably be in a sorority or fraternity…I wouldn’t even say it has anything to do with academic major.” Like Rachel, Sharlyne participated in programs for “minorities” in STEM, but this did not engender social and campus belongingness:

It’s hard being a minority here, especially my freshman year, sometimes I just felt like I was the only one…I came from a very diverse place, so, like being submerged into an old way, a culture that has…roots from 1870 or whatever—it can be really challenging figuring out your place at a school like this.

Black students constituted ~3% of undergraduates, inviting stereotype threat. As Sharlyne said, “some people might think, you know, African American students are here because [of] affirmative action.” Sharlyne reported, “I don’t really think about being female here. I think more about race…your race can kinda like shape your experience.” Though Rachel and Sharlyne disavowed gender as salient to their belongingness, we observe intersectional differences in the negative experiences Black students reported. In 2016, few organizations at MSU highlighted intersectional identities. MSU’s comparatively highly visible Black-identified resources (e.g., the Black Student Union) could explain why students like Sharlyne viewed their ethnoracial identity as the primary force shaping their college experiences. Black students’ stories add depth to our survey finding that Black women in STEM majors were less willing to return, despite non-significant findings for this group on belongingness measures. The experiences of STEM-focused Black women unwilling to return demonstrate how a hostile social context decreased their social and campus belonging to such an extent that satisfaction with academics could not make up the difference.

### Qualitative insights: Academic belonging

Supporting survey results that academic belonging is important to students when evaluating their college experiences, and that it varies intersectionally, interviews reveal the nuanced ways students assessed factors that influence these measures. We identify intersectional differences in academic belonging as a prominent explanation for willingness to return.

Interviewees mentioned enjoying their majors more following advanced classes, which facilitated increased skills and “getting to know” professors. Mary, a Latine woman in Engineering, rarely attended office hours her first year. But sophomore year, interactions with professors facilitated belongingness: “I’m really starting to get to know [my professors] and it’s like I go to them if I ever need help and I feel like that’s had a really positive effect on me.” Mary bemoaned her “bad GPA” (2.6 grade point average), but satisfaction with professors, the support she received, and the skills she gained balanced out dissatisfaction with her grades. In contrast, Neil, a White man in Finance, found his classes disappointing and low-quality. Not having to work hard, nor feeling he gained useful skills in his classes, motivated Neil’s regret in having chosen MSU.

Addie, a first-generation student from Vietnam pursuing a degree in the College of Business, was satisfied with MSU’s quality and reputation, but reported that “you have to get [a] good relationship with your professor” because networking was crucial for success. However, she saw herself at a disadvantage: “I feel like, students like me, I don’t get help from professor[s] that much…getting attention from people is hard, and, in my major, it’s very important to make the professors and companies to know you, remember you. So I think it’s a challenge…” An international and transfer student, Addie found professors less likely to reach out to someone like her; “It’s up to myself.” Rachel, the Black Engineering major profiled previously, reported similar feelings of stereotype threat: “my teacher in math is White…I feel like sometimes [professors] might think I’m automatically stupid.” Rachel attributed her fears to MSU’s “social environment:” specifically, the lack of faculty and student diversity raised prospects for unequal *academic* treatment.

Antonio, a Latine man in Engineering, experienced such negative faculty interactions that he considered transferring. Antonio received the typical advice to attend professors’ office hours to establish rapport. During one visit, however, the professor greeted Antonio, then audibly complained to a colleague, “Ugh, he’s always here!” Antonio was shocked; “I was just like, ooh, I, I just felt like that hurt.” Thinking he could not simply leave, Antonio went through the motions: “I asked her a question or whatever, got over the question and then I kinda just picked my stuff up and left.” Afterward, he changed concentrations within Engineering, and abandoned office hours: “I just stopped going…I completely blocked it off.” Despite his heavy involvement in organizations for Latine engineers, he cited the need for better community and support systems. Antonio worried he would lack strong letters of recommendation for graduate school because his relationships with professors were underdeveloped. If he could choose again, Antonio would pick a university “that is a little bit more, I guess, open to Hispanics.” Antonio’s negative faculty interactions outweighed his major and skill-development satisfaction, leading to worries about his post-college trajectory.

Previous research recommends undergraduate research as a means of building academic belongingness [60]; yet, interviews suggest that family income status may influence low-income students’ ability to participate. Undergraduate research opportunities were typically unpaid, and undertaken only when internships—often highly competitive—could not be secured. Though little research provides comparisons of internship relevance and availability across majors, it is clear that internships are expected, competitive and highly valued in some majors (e.g., Engineering, Business) and less common/less expected in others (e.g., Biology, English). At least one study found that for majors in Engineering and Business, it is more likely—as compared to social science and humanities majors—that a successfully completed internship will lead to a job offer [88]. Mary, the Latine Engineering major, applied for internships “left and right,” but companies “turned me away because my GPA was bad.” Mary found an unpaid research position through a faculty connection: “I’m kind of like one of his favorite students.” Mary was excited about doing research, but disappointed not to have applied internship experience, which Engineering respondents universally valued highly. Mary acknowledged that having her parents’ financial support made accepting a research opportunity feasible. Ricky, an Asian-American, first-generation Food Science major, dismissed his research experience; he had not “scratched the surface enough…need some job experience. ‘Cause even though I’ve done research, I need to do stuff outside [in] the field.” Ricky did not think research directly applied to his plans: working for a food industry conglomerate, while saving to open a restaurant. Mary’s and Ricky’s experiences suggest that undergraduate research, though a résumé-builder, may be inaccessible for low-income students, and was less valued as a career-builder than paid internships.

The experiences of Addie, Rachel, Mary and Antonio demonstrate why interactions with faculty and perceptions of academic support mattered for students’ satisfaction with MSU, implicating academic belongingness as an underlying explanation. Addie, Rachel and Antonio felt disregarded by faculty due to one or more marginalized identities. Mary’s connection with a professor garnered an unpaid research opportunity, which her family income status made feasible. Importantly, MSU has the power to address these limitations in ways that could boost students’ satisfaction and sense of belonging. For example, MSU could train faculty to improve their skills in working with underrepresented and marginalized students, and fully fund undergraduate research experiences. Absent such interventions, we found that race/ethnicity, family income, and other student characteristics influenced faculty interactions and academic experiences in complex ways, offering additional reasons why simultaneous membership in intersecting identity categories differentially affected sense of belonging at MSU.

### Qualitative insights: Working for pay and family income status

Interviews explored students’ paid work and family income status, following prior research finding that paid work can significantly slow down students’ progress. Seventeen interviewees (46%) worked for pay; seven total (19%) reported family incomes of $50,000 or less. The 17 employed students included all eight of those unwilling to return to MSU. In-depth questioning on this topic supplements survey findings suggesting positive connections between paid work hours and social and campus belonging, as well as willingness to return, but no relationship between this measure and academic belonging. Survey results did not find significant relationships between first-gen status, belongingness and willingness to return. NSSE does not ask participants to report family income, a limitation we also supplement with interviews; it is worth noting that four—half—of the eight interviewees *unwilling* to return to MSU reported family incomes of $50,000 or less. Interviewees broadly agreed (68%) that family income is a “factor increasing chances of success,” influencing “doing well in college” and “getting ahead in society.” Qualitatively examining family income and paid work experiences offers insight into how and why these factors may differentially influence belongingness and willingness to return, particularly among STEM majors.

Luis, Kyle, and Hayden, all Black men, related struggles due to low-income status, and their experiences implicated intersectional inequalities. Luis (Business), slowed down: “I took a semester off to work…I realized, crap, I can’t pay for this semester, I got to take this off.” Kyle (English) had financial difficulties despite his scholarship, loans, and paid work: “I actually had to cancel my meal plan ‘cause we didn’t have enough money.” Hayden (Sociology) reported that “good” unpaid internships were “interested in me,” but inaccessible:

[Income matters] just in terms of being able to afford to do things that others can’t. Like when I brought up the unpaid internship thing; some of these opportunities will look great on my résumé…one I saw was an unpaid internship for the White House…Even if they look great on our resumés, and open doors for us, and help us make connections, we [can’t]—there’s no way for us to take that opportunity.

Hayden additionally felt unwelcome in some social spaces:

…there are things [activities] that before I’m even thinking of joining, I have to think about, first of all, let’s say the class [status] of who’s being there. Like, I’ll want to know if my experiences would let me fit into that group, and sometimes I feel like with different groups they look at me and *they just think that I wouldn’t want to be there*, because of either my race or my [class] background; *I never know what it is*. (italics added)

Hayden could not know definitively whether his intersecting identities affected his social belongingness, but sensed “something” inhibiting his inclusion in student organizations and other social spaces. Hayden’s reciprocal hesitancy led him to question whether his experiences would “let me fit into that group;” that is, if others would perceive a low-income Black man as a valued contributor.

Interviewees also linked family income to the ability to access high-impact educational practices. Shane, a White man in Engineering, spoke dismissively of a classmate’s expensive study abroad, deeming it academically frivolous. Yet, Shane reflected, perhaps “the study abroad, like, ‘looks good’ [on a résumé]…having the ability to go out there and explore a little bit more…having money not be an issue.” Thus, family income status led to differential access to signature college experiences that “look good” to others, enriching belongingness.

Moreover, interviewees connected family income security, and associated freedom from the *necessity* for paid work, to lower stress, which also facilitated success. Hannah, a Multiracial woman in Engineering, expressed confidence: “[H]onestly, I know I’m going to make it through. I mean, I don’t really worry too much…I’m fortunate ‘cause my parents are paying for my school… So, I just gotta make sure I pass all my classes.” None of the six STEM-focused women interviewees with jobs worked more than 12 hours per week. Three specifically mentioned valuing “flexible” jobs, where they could cut back whenever school became “crazy busy.” Women interviewees who were STEM majors agreed: STEM success was incompatible with high employment hours.

Survey results showed no relationship between academic belonging and time spent working for pay. Yet, interviews with STEM-focused women suggest there may be intersectional nuance within this non-finding. That is, dominant groups in STEM appear to set the terms of STEM major engagement in ways that foment intersectional oppression, creating barriers for women and low-income students that differentially affect their satisfaction and sense of belonging. While Kyle worried about his scholarship’s GPA requirement and Luis had to take a semester off, Hannah’s parents paid tuition and rent, reducing stress and making work optional. Thus, income security helped compensate for factors otherwise negatively associated with willingness to return and belongingness, such as being a BIPOC student in STEM.

Hannah’s serenity sharply contrasts the stress inflecting the experiences of low-income students like Kyle and Luis. Hayden noted, “people who are financially well-off…that gives you greater agency…like we all come here, we all make mistakes, but some of us can’t come back from those mistakes like others can.” Higher-income status smoothed academic pathways, facilitated high-impact educational practices, reduced stress, and eased demanding STEM schedules. Low-income status inhibited academic, co-curricular, and social opportunities in a context that provided higher-income students with opportunities to utilize monetary resources to gain exclusive access to valued opportunities, such as unpaid internships and study abroad. Institutions have the power to enact policies and practices that can reduce the extent to which students’ economic resources matter for building academic, social and community belonging. For example, institutions can provide student scholarships for internships and study abroad, or directly fund co-curricular activities. To enhance social belonging, MSU could provide additional education and resources for student organizations, potentially addressing the “unwelcome” atmosphere Hayden reported. MSU lacked such supports; thus, the limitations experienced by underrepresented students were exacerbated at the intersections of marginalized identities, differentially affecting students’ sense of belonging.

## Discussion

Our mixed methods approach illuminates a range of challenges affecting undergraduates’ sense of belonging, as measured by expressed desire to return to MSU if given a chance to “do it all over again.” Our findings affirm prior research that when belongingness is missing, students express less satisfaction with their college experiences [6]. Furthermore, as predicted based on prior research findings, the extent to which these factors affected outcomes additionally varies by identity and other background factors [39–41]. That is, differences at the *intersections* of race/ethnicity, gender, family income and associated factors indeed matter for students’ willingness to return. Moreover, we found that willingness to return is influenced by major, with significant differences for STEM versus non-STEM majors, as well as nuanced differences when disaggregating by academic, social, and campus belonging. Taken together, our findings lend support to prior findings that (1) sense of belonging and satisfaction matter when it comes to students gaining benefits from their college experiences [6, 30, 31]; (2) sense of belonging is a complex construct, and results vary by the three types of belonging (academic, social, and community) identified by Nunn [7]; and that, *in some cases*, (3) women, BIPOC, and low-income students face greater barriers to developing a strong sense of belonging in college as compared with men, White, and wealthy students, and these issues are often exacerbated among students whose identities have been marginalized in STEM majors [19, 22, 65].

Our critical quantitative approach also reveals new findings, including some unexpected results that highlight the complexity—and necessity—of utilizing a critical and intersectional framework to explore college belongingness and satisfaction [30, 66–72, 77, 79]. We show how intersectional oppression matters for student experiences using a modeling strategy that uncovered inter- and intracategorical differences by race, gender, and academic major. Our study’s methods contribute depth and nuance, extending beyond previously reported quantitative patterns [e.g., 32, 35] to provide qualitative insight on the experiences of marginalized students, demonstrating *how* and *why* sense of belonging varied intersectionally. Our ability to untangle the three areas of belonging that Nunn [7] identifies—academic, social and community—revealed how belongingness in only one area (e.g., academic) was often insufficient for students with intersecting marginalized identities to persist in STEM majors and to express a willingness to return, providing a counterpoint to some previous survey-based results [23, 59]. We uncover significant gaps remaining in the goal of providing equitable outcomes at a public U.S. research university.

When assessing student satisfaction and sense of belonging, context of intersectional experiences mattered. Although STEM majors reported higher academic belonging and satisfaction per their willingness to return, this finding did not hold across all groups majoring in STEM. For example, women were more willing to return to MSU and expressed higher levels of sense of belonging. A complex array of positive and negative findings existed for ethnoracially minoritized students for each form of sense of belonging. One group that highlights these complex findings are Asian and Pacific Islander students and their experiences of social belonging at MSU. While our interaction term models found Asian and Pacific Islander *students* had lower levels of willingness to return than their White peers, Asian and Pacific Islander *students in STEM* held higher levels of willingness to return to MSU. The need to be attentive to the particularities of intersectional positions and experiences on campus are also exemplified by the lower levels of social belonging for Black men—regardless of academic major—and multiracial/other-race men in STEM degree programs. Among the social location models, across all ethnoracial student groups, when exploring the intersectional experiences and social locations by gender and academic major, no singular narrative fits our findings concerning student sense of belonging and willingness to return. These complex results add nuance to patterns found in some previous sense of belonging literature [cf. 6], and highlight the contributions of our mixed methods approach that attends to intersectional oppression. Our survey data provide the broader contours of campus inequalities, while qualitative inquiries plumb the depths of how and why ethnoracial-gender intersections mattered to students’ opportunity to build and activate a sense of belonging on campus—most poignantly, in interviews with Black men and women.

Qualitative results revealed important components of academic belonging included faculty-student interactions, perceptions of academic support, and a privileging of STEM degree programs and students over non-STEM students and their degree programs at MSU. Our analyses align with some previous findings, suggesting faculty responsiveness, mentoring and high impact practices like internships play an important role, particularly in STEM programs [20, 62, 89]. Perceiving better interactions with faculty increased belongingness. Interviews linked feeling ignored or disrespected by faculty with decreased belongingness, in some cases leading students to change majors. Thus, complicating previous belongingness research, we find that academic pathways among underrepresented and marginalized students may be linked to whether students gain a sense of community inclusiveness, including in their major [7]. Conversely, feeling that MSU supported students’ growth and development, including job-related skills acquisition, increased satisfaction and belongingness. Taken together, our quantitative and qualitative findings demonstrate that, particularly for BIPOC students and those subject to intersectional oppression due to multiple marginalized identities, satisfaction with academics did not always outweigh deficiencies in other areas of campus life shaping belongingness.

Our quantitative results suggest that willingness to return to MSU increases as paid work hours increase in on-campus jobs. This positive trend differs from prior research on working for pay while pursuing higher education [41]. Though this finding warrants future investigation, we suggest it may speak to how students are building connections with other peers working on campus alongside them, which then translates to increasing their sense of belonging. This somewhat unexpected finding is concerning because university costs continue to outpace inflation, despite many universities eagerly recruiting first-generation and low-income students. Despite uneven connections between each form of sense of belonging and willingness to return to MSU, first-generation status, and paid work hours in survey results, our interviews confirmed family income and associated factors as influential for students’ college trajectories, with STEM-focused women most adamant that working for pay more than 12 hours per week was incompatible with STEM success.

Furthermore, income and ethnoracial identity intersected to additionally shape belongingness. Our interviews with low-income Black men signal multiple ways intersections of marginalized identities link to more troubling experiences, less opportunity to participate in co-curricular activities, and a weaker sense of belonging. Universities serious about transforming the structure of opportunity and the cultural contours shaping belonging among BIPOC, low-income and first-generation students, particularly in STEM majors, should initiate visible, institution-level efforts to protect students from microaggressions and stereotyping, and support broad access to high-impact educational experiences such as study abroad regardless of income status. These efforts must include the ability to cultivate belonging with students’ intersectional experiences in mind, but also allow for holding the institution accountable for progress toward creating a more equitable, inclusive, and just campus [20, 79]. Our findings provide insight into college students’ complex lives in ways that may not come to light when utilizing singular methodological approaches. These complexities require mixed methodological approaches to uncover how and why institutional initiatives and experiences can lead to simultaneously differing student outcomes.

## Conclusion

### Limitations

Using available NSSE data hindered a full exploration of the intersectional differences in students’ satisfaction and sense of belonging. Smaller samples for some groups limited our ability to construct models that were race-gender specific (i.e., Black women, Latine men). Though we utilized some critical quantitative methodological approaches, additional data could assist with elucidating how satisfaction and sense of belonging differences reflect students’ complex intersectional experiences, particularly in STEM [19, 68, 69]. Moreover, while the age of the data utilized may be of concern, the recent global pandemic exacerbated previous findings that intersectional inequalities persist on college campuses and impact the degree pathways of students [90, 91]. Future research using more recent data from MSU can identify the extent to which students’ sense of belonging has changed, for whom it changed intersectionally, and how college campuses have adjusted their support for students from different social locations as we continue to understand the impact of the global pandemic on student degree pursuits and academic success. Additionally, while our modeling approach used marginal effects for comparisons across multiple groups, other modeling strategies can assist with clarifying and adjusting for multiple comparisons exploring intersectional differences among students.

Data limitations prevented us from fully examining differences among STEM majors. Women and BIPOC remain underrepresented in PEMC majors (Physics, Engineering, Mathematics, Computer Science), yet parity or a gap reversal has occurred in STEM fields like Biology and Psychology [44]. MSU’s concerns for protection of human subjects required institutional data on STEM-identified majors to be grouped by college. Thus, our results group students across science-focused colleges (e.g., biology, physics, math, and psychology are all housed in the same college at MSU) which might explain why some ethnoracial-gender outcomes observed in Table 4 (full sample) exhibit different patterns in Table 5 (STEM and non-STEM subsamples).

Our only quantitative measures related to family income were first-generation status and paid work hours in on- and off-campus jobs, limiting our ability to comprehensively explore the expected relationship between income status, satisfaction, sense of belonging, and other important social factors. Pairing our critical quantitative modeling strategy with interviews conducted on the same campus allowed us to further explore intersectional differences in family income and associated factors. Future work will examine longitudinal interview data gathered from the full sample of 113 MSU students. Each higher education institution has some unique features, but we expect that quantitative results concerning inequalities in patterns of satisfaction and belonging we uncovered at MSU are likely present at other research-focused, public institutions in the U.S. We utilized NSSE surveys that were fielded at MSU, but since NSSE’s validated survey questions are given in the same way at many other universities, we would anticipate some generalizability of our findings from this portion of our study. Our qualitative interview findings are less likely to be broadly generalizable. However, it is important to note that, in collecting interview data, generalizability was not our goal; rather, we sought to investigate the particularities of MSU’s context in greater depth.

### Future directions

While our quantitative analyses highlight differences in students’ sense of belonging across the three forms based on measures of institutional support, such belonging and willingness to return can be shaped by faculty interactions and research experiences that were not explored in our initial models. Previous research documents that underrepresented and marginalized students face barriers to research opportunities, or fail to fully benefit from them because of discriminatory experiences [51, 56, 58, 92]. In our interviews, students noted how they navigated research opportunities and preferred paid internships. Their experiences and stated preferences further complicate our understanding of the barriers and opportunities students encounter when pursuing STEM pathways; in particular, BIPOC interviewees’ responses suggested microaggressions and other negative faculty interactions can lead to disengagement. That is, micro-level experiences reveal the contours of macro-level marginalization and benign neglect. Further research will explore these possible connections both in subsequent interview waves and available survey data. Our findings here suggest that universities should assess the distribution and funding of internships and research opportunities, in conjunction with the quality of faculty-student interactions across student groups. Targeted funding for students—and training for faculty—could be strategic options for eliminating avenues that allow well-resourced students to accumulate advantages. Structural changes such as these may help reduce disparities and increase belongingness.

We know that policies designed to encourage achievement and persistence have varied effects, inflected by student identity and major [19, 63]. Future research must continue to examine these factors intersectionally, using mixed methods and longitudinal data to follow students’ progress over time. Our results here indicate the need for future institutional and quantitative research to afford the possibility of disaggregating broad panethnic categories such as “Asian/API” and “Latine” in order to be able to suggest targeted and specific interventions. Disaggregated data may better reveal how differential histories, immigration policies, and experiences of intersectional oppression are driving the remaining inequities we observe [93].

Examining college pathways at the intersection of two or more structures illuminates how systems of oppression are linked [66], thereby revealing the (re)production of beliefs, attitudes, and behaviors that are simultaneously gendered, racialized, and classed [70]. Structural changes are required to end practices that—consciously or not—support intersectional oppression by favoring White, upper-income men as the “default” STEM students in the U.S. Our research supports a growing body of evidence that institutions must actively build models of inclusion for underrepresented and marginalized groups to address inequitable and unjust practices, providing transformative mentoring and educational guidance that attends to intersectional oppression, in order to effectively support the next generation of women and BIPOC scholars [18, 20, 24, 58, 79]. Our mixed methods approach reveals how and why multiple factors related to college satisfaction influence students’ sense of belonging. Our interviewees had strong opinions about MSU’s social ecology, which BIPOC students viewed as White-centric. A thorough examination of MSU’s social life is out of scope here, but our future work will address this topic in greater depth. Academic leaders would do well to pay closer attention to how satisfaction with academic supports may not outweigh dissatisfaction with faculty, interpersonal relations, and/or access to high-impact experiences, maintaining inequalities in belongingness along intersections of race/ethnicity, gender, income and major.

## Supporting information

S1 Table Table S1: Control measures for intersectional fixed effects models of academic, social, and campus belonging and willingness to return to MSU with interaction terms and social location. (DOCX)

S1 File Appendix A: Qualitative semi-structured interview schedule. (DOCX)

S2 File Appendix B: Qualitative codebook. (XLSX)

S3 File Appendix C: Author positionality statements. (DOCX)
