# Supplementary material for: “Figuring out your place at a school like this:” Intersectionality and sense of belonging among STEM and non-STEM college students
Source: PLoS One. 2024 Jan 10;19(1):e0296389. doi: 10.1371/journal.pone.0296389 (PMC10781048; doi:10.1371/journal.pone.0296389)
Supplement: S1 Table — (DOCX) [file pone.0296389.s001.docx]

Table S.1. Control Measures for Intersectional Fixed Effects Models of Academic, Social, and Campus Belonging and Willingness to Return to MSU with Interaction Terms and Social Location

| Variable | Academic Belonging | | | Social Belonging | | | Campus Belonging | | | Willingness to Return | | |
| --- | --- | --- | --- | --- | --- | --- | --- | --- | --- | --- | --- | --- |
|  | AME | SE | *p* | AME | SE | *p* | AME | SE | *p* | AME | SE | *p* |
| Academic year | -.062** | .018 | .001 | -.044** | .016 | .008 | -.047** | .017 | .007 | .003 | .017 | 0.863 |
| Grades | .100* | .047 | .034 | .118* | .046 | .010 | .097* | .047 | .039 | .075 | .050 | 0.140 |
| Transfer student | -.127 | .069 | .068 | -.203** | .064 | .002 | -.149* | .068 | .031 | -.043 | .070 | 0.538 |
| First-generation student | -.007 | .064 | .904 | -.028 | .059 | .641 | -.064 | .063 | .310 | .018 | .060 | 0.758 |
| Hours worked on-campus | .024 | .016 | .121 | .060*** | .015 | .000 | .066*** | .016 | .000 | .051** | .015 | 0.001 |
| Hours worked off-campus | -.022 | .016 | .191 | -.009 | .016 | .575 | -.010 | .016 | .534 | -.005 | .015 | 0.730 |
| NSSE Year | -.133*** | .035 | .000 | -.154*** | .032 | .000 | -.037 | .034 | .272 | -.075* | .032 | 0.022 |

Notes: Analyses used imputed data and contained 3,392 cases each. Control measures not shown include academic year of students, reported grades, whether the student transferred to MSU, first-generation status, hours reported working for pay on- and off-campus, and year of survey. White students, women, and non-STEM majors are reference categories. The control measure findings were similar for both sets of fixed effects models (interaction terms and social locations) as the approach taken to including race/ethnicity, gender, and academic major changed to elucidate intersectional differences in sense of belonging and willingness to belong, yet the approach to including control measures remained the same. “AME” represents average marginal effects. “SE” represents standard errors.

* *p*<.05; ** *p*<.01; *** *p*<.001
