# Supplementary material for: “Figuring out your place at a school like this:” Intersectionality and sense of belonging among STEM and non-STEM college students
Source: PLoS One. 2024 Jan 10;19(1):e0296389. doi: 10.1371/journal.pone.0296389 (PMC10781048; doi:10.1371/journal.pone.0296389)
Supplement: S1 File — (DOCX) [file pone.0296389.s002.docx]

**Appendix A**

**Qualitative Semi-Structured Interview Schedule**

| **Background** | - Age - Race/ethnicity (when you are asked, such as filling out a form) - Race/ethnicity (at home or among friends) - Born in the United States? - IF NO: Keeping in mind that your responses are confidential and you can opt out of responding, what is your current citizenship status? - Probe: are you currently working towards gaining citizenship in the US? - Please tell me a bit about your life growing up: your siblings, your parents, and the places you have lived. - What was the highest grade/degree your parents completed? - IF COLLEGE: what college?   - Probe: [if R says parents did not attend] Did they participate in any post-high school training of any kind? - Occupation(s) of parent(s)   IF ANY SIBLINGS: What are your siblings doing now?   - Do your parent(s)/guardian(s) own or rent their residence? - Can you estimate your parents’ income level?   - Probe: [Show card] Here are a number of income ranges. Would you say your parents’ combined income is (less than 25k, between 25-50k, between 50-80k, between 80-150k, between 150k-250k, more than 250k?) |
| --- | --- |
| **College Decisions & Academics** | - Please tell me all about how you ended up here at [this college]. - Probe: When did you start looking at colleges, what was that like? How many colleges did you apply to? How did you decide to come here? Why did you want to go to college? - What is your major? Is it STEM major? Is it a science major? How would you compare your major to other similar majors? How would you rank your major compared to other similar majors in your college? How did you decide on that major? - Probe: talked to a counselor, faculty about that major? - What year are you in?   - Thinking realistically, how much longer do you think you have before graduation? - What courses are you taking this semester? How many credits is that? Is that a typical load for you? - What is your GPA right now? - What is your favorite class that you’ve taken so far? Why? - What has been your least favorite class? Why? - What has been your most difficult class? Why? - Do you think you’ll have any difficulty completing college? IF SO: tell me about that.   - How are you paying for college? Probe: what kind of financial aid, loans, etc. - Do you ever study/do assignments with others, such as in a study group? If so, how was the study group formed?   Do you have a documented disability, including any learning disabilities?  Have you ever needed an accommodation at school?  Did you have accommodations in high school?  Do you use accommodations at [this college]?  Tell me about your experiences in asking for accommodations  - Do you believe that you have all the accommodations you need? |

| College Life | - **What does it mean to be a student at [this college]?** - Why do you think people go to this college? What draws students here?   - Do you think this was a “tough” school to get into?   - What are the pros and cons of attending [this college]?   - Before you came here, did you know anyone who had gone to [this school]? Who? Did they share information about the school with you? - What is your living arrangement right now?   - Probe: Do you or does someone else own your residence, or do you rent? [for example, did R’s parents buy a house/condo for them to live in during college]   - Have you had other places to live since coming to college?   - [If R lives off campus] How did you find your living space? - Do you currently have a job? Where do you work? (on-campus, off campus, work-study) - How many hours per week do you work? - What do you do with the money you earn? Probe: are you expected to work? - What clubs or organizations do you participate in? About how many hours per week do you spend on each of these activities or with members of these orgs? - Probe: Sports, Greek life, clubs, Military/ROTC, etc. - What other academic activities have you participated or plan to participate in?   - Probe: Study abroad, internships, research with faculty, independent studies or research, conference presentations or publications? - Do you participate in any activities not related to campus life?   Probe: Church activities, service learning, etc.   - How did you choose those activities? What do you get out of them? Are there any activities, clubs or orgs that you have quit? - If you could do it again knowing what you know now, would you come to [this college]? Why or why not? |
| --- | --- |
| **Goals** | - **What are your educational and career goals?** - What steps so you think are necessary to achieve this goal? What steps have you taken/will you take to reach this goal? - About how long have you had this goal? (Probe: since you were a kid, since HS?) Have your goals changed over time? If so, why and how did it change? - How did you come up with the idea? Did anyone else influence your decision or suggest this goal to you? - Do your friends or family members have similar goals? Are there a lot of people like you who have achieved this kind of goal? Are your friends from home also going to college? - What concerns or fears do you have about achieving your goals? - Has there been a teacher, professor or staff member who helped you with your goals or just helped keep you on track? How did that person help? - In your life, has there been a person or experience that has been influential in helping you focus on your goals? Please tell me about that person or experience.   - How do you define success? - What factors increase your chances of success? - What do you see as barriers to your success? |
| Family | - **How has your family influenced your educational aspirations and expectations?** - Do your parents talk with you about schoolwork or studying? - Do your parents ask to see your papers, exams, or other class materials? - Do your parents ask to know your GPA? - Have your parents ever talked to or contacted your instructors here?   - [If so,] What do they talk to your instructors about? - Do your parents talk to you about your college experience more generally?   - [If so,] Specifically, what about college do you talk with your parents about? - What goals do your parents have for you?   - Probe, if too general: Have they mentioned anything more specific than that, like a particular career or personal goal? - How long have they had this/these goal(s) for you? - Have they helped you toward achieving this goal? How, specifically? - What reasons do your parents give for why this is an important goal? |
| Friendships and Peer Groups | - **What are friendships like at [this college]?** - How would you characterize your friendship or peer group? Do you have one main group of friends, or several? (If several): What separates the groups? Why don’t all of you get together? - How do you think other people would characterize your group of friends? - Do you think your group of friends is similar or different to other groups on campus? Do you feel like you “fit in” here? IF YES: why do you think so? what about you makes you and this college a “good fit?” IF NO: Why not? What does it take to “fit in” at [this college]? - What advice would you give to someone like you who is considering coming to [this college] about fitting in here? Are there any challenges you would warn them about? What about benefits to being someone like you here?   - Probe: (if they ask, what do you mean by “someone like you”) Someone your race/ethnicity, gender, income group - Do people from other friendship groups ever treat you differently? - Are most of the friends you hang out with at school the same race/ethnicity as you?   - About what percentage would you say are the same race/ethnicity?   - Is this the same or different than your friends back home?   - What kinds of social media to you use? What do you use social media for? Do you use different social media platforms for different reasons? Are your friendship groups different on social media than they are in your daily life? |
| Gender & College; Race/ethnicity & College; Income & College | - **What is the best way of getting ahead in American society?** - What can prevent students from doing well in college?   -Probe: In your opinion, what should be done to solve that/those problems?   - Are there any people or groups that have an advantage when it comes to doing well in college?   -IF NO: So everyone has the exact same chance of doing well in college?  -IF YES: Please tell me more about that. Which groups or people would you say have an advantage? Why do they have an advantage?   - Are there any people or groups that have a better opportunity to get ahead today than in the past?   -IF NO: So there are no people or groups that have better opportunities today as compared with in the past?  -IF YES: Please tell me more about that. What makes you think so? How does knowing that make you feel (good, bad, it’s unfair, etc.)?   - Do you think that people of all races have an equal chance to do well at [this college]? Why? Why not? - Do you think that people of all genders have an equal chance to do well at [this college]? Why? Why not? - Do you think that people with disabilities have an equal chance to do well at this college? Why? Why not? - Do you think this college is accessible for people with disabilities?   What about income level? Do you think people of all income groups can do well at [this college]? Why? Why not?  What about class status? Probe: Is class status different than income level? Why do you think so? Does class affect how well you can do at [this college] differently than the amount of money you have? Why or why not?  Do you think students with certain political beliefs one way or another (e.g. conservative or liberal, etc.) have a harder time fitting in at this school than others?   - Which people have the hardest time doing well at [this college], and why do you think so? Earlier I asked about fitting in—is there any relationship between fitting in and doing well in college? - What is it like to be [insert respondent’s stated race/ethnicity] at this school? - What is it like to be [stated gender] at [this college]? - What is it like to be [stated income level] at [this college]? - What is it like to be [a person with a disability] at this college? |
| Conclusion | Is there anything else that we haven’t covered that would be important for me to know about life at [this college]?  Are there any other challenges you face or benefits you get from being who you are that I haven’t asked about?  Do you have any questions for me? |
