# Supplementary material for: “Figuring out your place at a school like this:” Intersectionality and sense of belonging among STEM and non-STEM college students
Source: PLoS One. 2024 Jan 10;19(1):e0296389. doi: 10.1371/journal.pone.0296389 (PMC10781048; doi:10.1371/journal.pone.0296389)
Supplement: S3 File — (DOCX) [file pone.0296389.s004.docx]

**Appendix C**

**Author Positionality Statements**

Sarah M. Ovink identifies as a middle-aged, white, highly educated, high-income, Ph.D.-holding sociologist, who is the daughter of college-educated parents. These identities afford me a constellation of privileges that could negatively affect my ability to empathize and create trusting connections with the college-aged interviewees included in this study. Even so, I had the experience of being guided away from pursuing a STEM major in my first year of college, despite my childhood ambition of pursuing a career in the natural sciences. Other experiences navigating sexism are dotted throughout my life. These experiences, and the knowledge that women and members of other historically marginalized groups are still underrepresented in the academy, has led me to focus much of my career activities on uncovering and seeking to address remaining intersecting barriers to equitable college-to-career trajectories in STEM and related social sciences. Whenever possible, I approach data collection and analyses with a team of graduate and undergraduate researchers whose life experiences are different than my own, which enriches our analytical project. Nevertheless, I acknowledge that my positionality, assumptions and biases may inflect my analyses, however unintended.

W. Carson Byrd identifies as a white, highly educated man who experienced his family’s transition from working class to middle class status. My educational experiences were marked by the policies of busing and white backlash to it, racialized and classed tracking in schools, “small town politics” that intermeshed social class and racialization into affording more opportunities for a small segment of children and families at the expense of others, and the denigration of friends and peers who identify as LGBTQIA+ trying to be acknowledged as members of a campus community, among other situations that have led me to examine how schools and colleges can simultaneously operate as engines of opportunities, inequalities, and injustice. I approach this work through connecting critical frameworks with quantitative and computational methods to clarify how opportunity, inequality, and injustice can exist within the patterns of data. Collaboration is important to build community and combat the intersectional injustices that can exist in our schools, colleges, and society as a whole, but also to better situate the research to confront assumptions and biases that shape analyses and interpretations. Despite such efforts, my contributions to this research may reflect unintended assumptions or biases.

Megan Nanney identifies as a white non-binary, feminine and queer person, who comes from a middle-class heteronormative family and whose parents are first generation college graduates. Yet, it seems futile to “string” my identifiers together with commas and expect that I have engaged with my positional relationships fully [1]. As such, I recognize that I grew up with the capital—human, social and economic—to understand how to easily navigate systems of higher education, and the privilege to do so successfully. But I also grew up in a system where I was discouraged from the STEM pipeline *because* of my positionality as a feminine person and currently struggle with the biases and inequities we write about as an employee in the tech field. As I conducted interviews with participants, I found that I leveraged this duality to empathize, develop trust, and form connections with students across the spectrum of experiences.

Abigail Wilson is a US-born white female identifying adult. During the time that this paper was written, she was an undergraduate student experiencing a similar context to that of interviewees. She acknowledges that her position as a student and now alumna influenced her positionality in this research. Additionally, she acknowledges that her experiences as a first-generation college student may have created a biased understanding of research participants’ upbringings and collegiate experiences.

1. Tillapaugh D, Nicolazzo Z. Backward thinking: Exploring the Relationship among Intersectionality, Epistemology, and Research Design. In: Mitchell Jr. D, Simmons CY, Greyerbiehl LA, editors. Intersectionality & Higher Education: Theory, Research, and Praxis. New York, NY: Peter Lang; 2014. p. 111–22.
